# Fascia Iliaca Compartment Block Efficacy in Resource-poor Emergency Departments

**DOI:** 10.5811/cpcem.2018.9.39208

**Published:** 2018-09-28

**Authors:** Daniel St. Louis, Kenneth V. Iserson, Nicolas Forget

**Affiliations:** * Georgetown Public Hospital, Department of Accident and Emergency Medicine, Georgetown, Guyana; † University of Arizona, Department of Emergency Medicine, Tucson, Arizona; ‡ Vanderbilt University, Department of Emergency Medicine, Nashville, Tennessee

## Abstract

**Introduction:**

Although the fascia iliaca compartment block (FICB) seems to be an ideal technique for femoral neck and shaft fractures occurring in resource-poor settings, it has been unclear how effective it is when used by emergency physicians (EP) with little training in the technique, using equipment, medications and methods that differ from those commonly available in developed countries. This series was designed to demonstrate that EPs in a resource-poor setting can provide effective analgesia for femur fractures with anatomic landmark-guided FICBs, clinician-compounded lidocaine-epinephrine (1:100,000), and a standard injection needle.

**Methods:**

Over a three-month period, patients ≥12 years old presenting to the emergency department with hip or femur fractures and a Likert visual analogue scale >4 had an EP-administered FICB. EPs used a standard intramuscular needle and a lidocaine-epinephrine solution they compounded at the bedside and located the injection site using only anatomic landmarks. EPs evaluated the patient’s pain level at 30 minutes and at two hours post-FICB. We also reviewed articles since 2016 that describe the FICB.

**Results:**

We enrolled a non-consecutive sample of 10 patients in the case series. Five had femoral neck (hip) fractures and five had femoral shaft fractures. All patients had a reduction in their pain levels after the FICB. On average, the block took effect about three minutes after injection. At 30 minutes all patients reported clinically meaningful pain reduction. The analgesic effect of the compounded agent lasted approximately 200 minutes. No adverse effects were reported. No published journal articles about FICB since 2016 were from resource-poor settings, and only one was from a developing country.

**Conclusion:**

This series suggests that the FICB is effective even when performed with the minimal materials that are usually available in resource-poor settings. Methods such as this, which use simplified clinical tests and techniques applicable in resource-poor settings, can assist global emergency medicine (EM). We can assist global EM by similarly finding methods to simplify useful clinical tests and techniques that can be used in resource-poor settings.

## INTRODUCTION

Patients with femur and hip fractures commonly present to emergency departments (ED). Promptly providing analgesia reduces the significant physical and psychological effects on patients. It speeds their operative management, lessens the length of hospitalization,^1^ and decreases opiate-induced complications, especially in the elderly.^2^ Yet only 2% of elderly patients with fractures receive adequate analgesia.^1^

Building on Sharrock’s accidental discovery of the fascia-iliaca compartment block (FICB) in 1980,^3^ BJ Dalens^4^ popularized its use and Godoy-Monzon^5^,^17^ introduced it into the emergency medicine (EM) literature in 2007. The FICB offers a safe, inexpensive, rapid and effective analgesic for patients with hip fractures.^5^ A single injection of anesthetic solution into the fascia iliaca space spreads to block the femoral, lateral femoral cutaneous, and obturator nerves, providing anesthesia to the hip, femur, and, often, the knee.^6^,^7^ The FICB’s safety stems from the needle insertion site’s location being distant from the femoral triangle (artery, vein, and nerve), which minimizes the risk of intravascular injection and nerve injury.^1^, ^6^

While numerous publications have demonstrated the block’s efficacy in relieving pain for patients with acute femur fractures, the methods relied on equipment and medications not available in most facilities outside the developed world and few have originated from resource-poor facilities in developing countries. Resource-poor settings, which often have only parenteral opiates to provide analgesia after acute fractures, are where FICBs may prove most useful. This paper reviews recent publications that describe FICB settings and techniques and uses a case series from a resource-poor hospital ED in a developing country to demonstrate the block’s efficacy using basic equipment, inexpensive and readily available medications, and novice practitioners.

## METHODS

### Setting and Population

The convenience study sample was patients ≥12 years old with moderately and severely painful (4/10 on the Likert visual analogue [VAS] pain scale) injuries to the hip and femur. Patients were excluded if they had a history of allergies to local anesthetics; a history of femoral bypass surgery; signs of an infection at the FICB injection site; a bleeding diathesis (known history or currently evident); or a history of second- or third-degree heart block. EM faculty and resident physicians performed all the blocks. The Medical Advisory Council (equivalent to an institutional review board [IRB]) approved this study. All participants or their surrogate decision-makers provided informed consent.

### Protocol

Prior to the study, participating emergency physicians (EP) attended a short teaching session, received a printed protocol with anatomical illustrations, and were shown the procedure for compounding the lidocaine-epinephrine mixture. After identifying appropriate patients with a VAS pain scale ≥4, the EPs obtained informed consent. They then completed a neurovascular examination of the affected limb and prepared the lidocaine-epinephrine 1:100,000 solution (1 milligram [mg] =1 milliliter [mL] 1:1000 epinephrine/100 mL lidocaine). Compounding the solution at the bedside, they added 0.2 mL of epinephrine (1:1000) to 20 mL of lidocaine 1% (10 mg/mL). They aspirated the mixture into a sterile syringe attached to a 21-gauge, 2-inch intramuscular injection needle.^5^,^8^

**CPC-EM Capsule**

What do we already know about this procedure?

Fascia Iliaca Compartment Blocks (FICB) are ideal for providing analgesia to emergency department (ED) patients with femoral fractures but are rarely used in resource-poor settings.

What makes this case series reportable?

Emergency physicians in a resource-poor setting successfully did FICBs using only anatomical markers, clinician-compounded analgesic and standard injection needles.

What is the major learning point?

FICB blocks can be successfully performed (clinically meaningful pain reduction within 30 minutes) with the minimal equipment and materials present in any ED.

How might this improve emergency medicine practice?

This study (the first from a developing country since at least 2016) should encourage clinicians in similar settings to use the FICB despite lacking high-level resources.

With the patient supine, the inguinal ligament was identified, the femoral artery palpated, and the skin cleaned with iodopovidone. The needle was inserted perpendicular to the skin at a point one centimeter (cm) below the juncture of the lateral and medial 2/3 of a line from the pubic tubercle to the anterior superior iliac spine (Image). Using the loss-of-resistance method, the needle was inserted until a loss of resistance was first felt as it passed the fascia lata and again when the fascia iliaca was pierced (often described as two “pops”). After aspirating to exclude intravascular placement, the mixture was injected into the fascia iliaca compartment. All patients received a standard 20 mL dose (200 mg lidocaine) of this mixture. Each patient’s pain level was re-evaluated 30 minutes and two hours after FICB administration.

### Literature Review

We searched PubMed for publication dates between 2016 and mid-May 2018 using the following search strategy: “Best Matches” for “fascia iliaca block” OR “iliofascial block”: [“fascia iliaca block”[All Fields] OR (iliofascial [All Fields] AND block [All Fields])]. The papers were then culled for non-review papers in English that had information describing their setting and preoperative-analgesia FICB protocol using a single (non-continuous catheter) injection for acute femur fractures.

## RESULTS

Ten patients aged 12 to 93 years (median 59 years) had FICBs using bedside-compounded lidocaine-epinephrine. Five patients were males; five had femoral neck and five had femoral shaft fractures. Seven EPs performed the blocks: five residents and two faculty. All seven initially performed the block for the first time. Medication preparation and block performance generally took less than five minutes. Repeat pain levels were measured at 30 minutes and 120 minutes post-block. All patients experienced clinically relevant pain reduction post-block at both time points. VAS pain scores averaged 7.1 (range 5–9) on presentation, 2.7 (range 0–4) at 30 minutes post-block, and 0.6 (range 0–2) at 120 minutes. The average duration of analgesia was 200 minutes. No adverse events were reported from the blocks. Most study patients were admitted to the operating room (OR) or ward before the block lost effect. For the few who remained in the ED, an additional FICB was administered. One patient refused to be transferred to the ward before he received a second FICB.

We identified 10 articles using the PubMed search. One was in Chinese, one described a femoral nerve block, and one had insufficient information. The Table contrasts the remaining articles with the current case series.

## DISCUSSION

Since 2016, all journal articles describing the use the FICB for analgesia after acute fractures have originated from resource-adequate facilities; all but one are from developed countries. In contrast, the current case series is from a resource-poor teaching hospital in a developing country, a situation in which the FICB is particularly useful. Resource-poor settings have less monitoring available for patients who have been given mind-altering analgesics, surgery may be significantly delayed due to a lack of staff or equipment, and inpatient admission delays may require EPs to repeatedly administer analgesia over several days. Yet clinicians in these facilities may hesitate to do FICBs without the equipment and medications most commonly described for the procedure. We conducted this case series to demonstrate that the FICB can be performed successfully with limited equipment, medication, and training.

### Reviewed Cases

Of the seven PubMed articles that met the criteria, only one was from a developing country.^9^ Based on website descriptions, none would be considered a resource-poor facility. In contrast, Guyana is a developing country and the ED routinely lacks basic medication and equipment, including those typically described to perform the FICB.

### Ultrasound Use

The loss-of-resistance FICB using anatomic landmarks does not require ultrasound guidance, since its safety relies on the distance of the thigh’s major neurovascular structures from the injection site. Neither the study that first described the FICB nor the initial studies demonstrating its utility for acute fractures in EDs used ultrasound guidance, although they had high success rates.^4^,^5^,^17^ Despite that, many publications that describe the procedure now advocate the use of ultrasound (or neurostimulators in the OR), with three of the recent articles describing its use. While our ED has an ultrasound machine, it does not have the linear transducer commonly recommended for use with the FICB. We therefore used the anatomic landmark, loss-of-resistance (or “two-pop”) technique for our blocks.

### Needle

A common recommendation for performing the FICB is to use a blunt 21G or a Tuohy (slightly curved epidural) needle to more easily recognize passage through the tissue planes. Three of five articles described using a Tuohy needle, while another used an echogenic needle with ultrasound guidance. In our setting, neither recommended needle was available. We successfully used an intramuscular injection needle.

### Medication

As the reviewed articles demonstrated, several long-acting local anesthetics work well for single-injection (as opposed to catheter-infusion) FICBs. Cost and logistics prevent our ED from stocking anything but plain lidocaine. To produce long-acting anesthesia, we added epinephrine to lidocaine, often doubling its anesthetic duration.^18^ For simplicity and safety, all patients received a standard 20mL dose (200mg lidocaine) of this mixture, which is less than the average maximum dose for a 12-year-old child (41kg=287mg), for an average adult (70kg=490mg), and for an elderly or renal-hepatic compromised patient (55kg=385mg/2=193mg).

### Expertise, Specialty and Location

No inexperienced clinicians in the reviewed publications performed FICBs, and only two had EPs doing the block. As in our setting, clinicians in resource-poor EDs often must perform novel techniques based on written, oral, or video descriptions. The blocks performed by our ED staff and resident physicians were successful, although they had learned the procedure using written and oral descriptions.

## LIMITATIONS

This study had two major limitations: only 10 patients met the study criteria; and the IRB did not permit sham injections for a control group. Nevertheless, the high success rate our EPs had with the FICB using inexpensive and readily available materials is consistent with the results in studies with the normally recommended equipment and medications.

## CONCLUSION

EM faculty and residents in a resource-poor ED successfully reduced the pain from femoral fractures by performing FICBs using only anatomic landmarks, physician-compounded lidocaine-epinephrine, and a standard injection needle. This demonstrates that physicians in resource-poor settings can safely and quickly perform the FICB with little training, using the equipment and medications they typically have available. Using the FICB in resource-poor, often rural settings also reduces risks related to opioid use and provides an excellent method to ease the pain of patients transferring to higher levels of care.

Documented patient informed consent and/or Institutional Review Board approval has been obtained and filed for publication of this case report.

## Figures and Tables

**Image:**
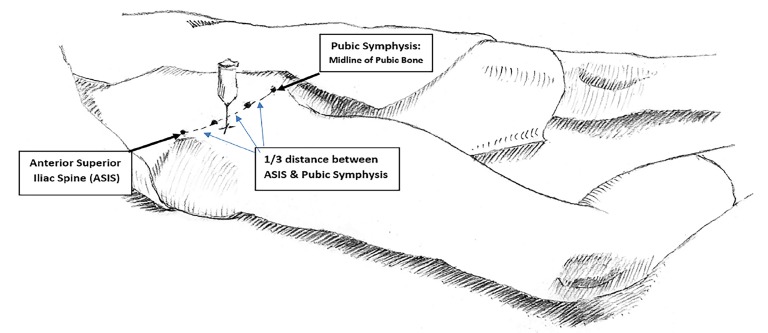
Anatomic landmarks for fascia-iliaca compartment block needle placement: one centimeter inferior to the point on a line joining the anterior superior iliac spine (ASIS) and the pubic symphysis.

**Table:** Comparison of current case series with recent (2016–2018) publications.

Reference number	Developing country?^9^	Resource-poor conditions?	Ultrasound?	Tuohy (epidural) needle?	Compounded anesthetic?	ED/EP?
10	Spain (NO)	NO	NO	Tuohy	Levobupivacaine 0.25% 0.3mL/Kg	YES/NO
11	USA (NO)	NO	YES	NS (Not specified)	YES. 0.5% ropivacaine + 2% lidocaine w epi	OR(Operating room)/NO
12	Bangalore, INDIA (YES)	NO	NO	Tuohy	0.375% ropivacaine; 30mL	OR/NO
13	Portugal (NO)	NO	YES	NO (Echoplex echogenic needle)	YES. 1.3% mepivacaine + 0.5% ropivacaine	OR/NO
14	UK (NO)	NO	NO	NO (Spinal needle)	Levobupivacaine 0.25%; 30 or 40 mL	YES/NO
15	UK (NO)	NO	NO	Tuohy	Levobupivacaine 0.25% + saline	YES/YES
16	Romania (NO)	NO	YES	NS (Not specified)	≤40 mL 0.5% Ropivacaine	YES/YES
	[This Series’ Country] (YES)	YES	NO	NO	1% Lidocaine + Epinephrine	YES/YES

*ED*, emergency department; *EP*, emergency physician; *USA*, United States of America; *UK*, United Kingdom; *mL*, milliliter; *Kg*, kilogram.
